# Design of Membrane Active Peptides Considering Multi-Objective Optimization for Biomedical Application

**DOI:** 10.3390/membranes12020180

**Published:** 2022-02-02

**Authors:** Niels Röckendorf, Christian Nehls, Thomas Gutsmann

**Affiliations:** 1 Division of Mucosal Immunology and Diagnostics, Research Center Borstel—Leibniz Lung Center, Priority Area Chronic Lung Diseases, Member of the Airway Research Center North (ARCN), German Center for Lung Research (DZL), Parkallee 22, 23845 Borstel, Germany; 2 Division of Biophysics, Research Center Borstel—Leibniz Lung Center, Priority Area Infection, Parkallee 10, 23845 Borstel, Germany; cnehls@fz-borstel.de (C.N.); tgutsmann@fz-borstel.de (T.G.); 3 Center for Structural Systems Biology (CSSB), Notkestraße 85, Building 15, 22607 Hamburg, Germany; 4 Kiel Nano, Surface and Interface Science KiNSIS, Kiel University, Christian-Albrechts-Platz 4, 24118 Kiel, Germany

**Keywords:** membrane active peptide, peptide design, multi objective optimization, cell penetrating peptide, antimicrobial peptide, membrane activity assay

## Abstract

A multitude of membrane active peptides exists that divides into subclasses, such as cell penetrating peptides (CPPs) capable to enter eukaryotic cells or antimicrobial peptides (AMPs) able to interact with prokaryotic cell envelops. Peptide membrane interactions arise from unique sequence motifs of the peptides that account for particular physicochemical properties. Membrane active peptides are mainly cationic, often primary or secondary amphipathic, and they interact with membranes depending on the composition of the bilayer lipids. Sequences of these peptides consist of short 5–30 amino acid sections derived from natural proteins or synthetic sources. Membrane active peptides can be designed using computational methods or can be identified in screenings of combinatorial libraries. This review focuses on strategies that were successfully applied to the design and optimization of membrane active peptides with respect to the fact that diverse features of successful peptide candidates are prerequisites for biomedical application. Not only membrane activity but also degradation stability in biological environments, propensity to induce resistances, and advantageous toxicological properties are crucial parameters that have to be considered in attempts to design useful membrane active peptides. Reliable assay systems to access the different biological characteristics of numerous membrane active peptides are essential tools for multi-objective peptide optimization.

## 1. Introduction

The smallest self-sustaining biological entities, colloquially simply called cells, divide into procytes that constitute archaea or bacteria and eucytes that are found in fungi, plants, and animals. Cells isolate their interior from the outside world by a protective cover, the core unit of that shield consists of a lipid bilayer called a membrane. The integrity of the membrane is an indispensable prerequisite for cellular function, biological cells ensure thorough supervision of the transition of material that permeate through their membranes by keeping them faultless.

In respect of this fact, an important feature of the cellular membrane is its semi-permeability; the majority of molecules are unable to cross the membrane barrier but some molecules are capable to enter or exit the cell. Basic mechanisms in membrane transport events include passive transcellular diffusion processes and active transport events usually mediated by carrier molecules [1,2]. Investigation of the molecular properties of compounds able to percolate lipid bilayers show that molecular size and lipophilicity of the materials are key aspects in passive diffusion events [3,4]. Compounds which are small in molecular size are able to surpass lipid bilayers via channels or diffusion, but the larger the molecules the slower the uptake processes unless active transport is taking place.

The integrity of lipid membranes of eukaryotic cells can be affected by toxic peptides produced by bacteria; contrariwise antimicrobial peptides produced by host cells directly influence the stability of prokaryotic target membranes [5]. In many cases, the surface charge of these membranes is decisive for sensitivity or resistance against the respective peptides. Most bacteria are highly negatively charged due to lipopolysaccharide (LPS) [6] structures in the outer leaflet of the outer membrane of Gram-negative bacteria. Phosphatidylglycerol (PG) in the membrane of Gram-positive bacteria mediates similar characteristics. However, more specific target structures for interaction with compounds affecting bacterial membranes, such as lipid II [7] are well known.

Peptides that are able to interact with lipid membranes and to manipulate their integrity by translocating through, disrupting or binding to, and fusing with the bilayer are called membrane active peptides [8]. In order to utilize these peptides in medical application, it is necessary to optimize their membrane activity and to overcome unwanted effects, such as toxicity or proteolytic degradability [9]. According to their mode of interaction, membrane active peptides can be divided into subclasses, such as CPPs that act as membrane transporters while maintaining membrane integrity and AMPs that act as membrane disruptors. Nevertheless, physicochemical and structural characteristics in both of these classes of membrane active peptides are similar; therefore, AMPs in some cases are active as CPPs as well and vice versa [10]. The membrane activity of both CPPs and AMPs is affected by differences in the composition of procytic and eucytic membranes and only to a lesser extent by the peptides structure. Sterols are missing in prokaryotic cell envelops and the share of anionic lipids is higher in this kind of membrane. These parameters directly influence membrane affinity and destabilization potential of membrane active peptides. Discrimination of the classes of CPPs and AMPs is therefore founded on a historical basis rather than on defined functional differences of peptides attributed to one of the above classes [11].

## 2. Cell Penetrating Peptides

The translocation of larger biomolecules, such as peptides, proteins, or nucleic acids through lipid membranes is limited. To overcome this limitation, a multitude of techniques were elaborated to force cells to ingest macromolecular materials. Biological tools to achieve this include the usage of viral vectors and physical methods to transfect cells range from electroporation to microinjections. Chemical agents that facilitate cellular entry include detergents or membrane active peptides. CPPs are a class of membrane active peptides that were discovered in the late 1980s when it falls into place, that some particular proteins are able to translocate through cellular membranes [12,13] and that this capability sometimes is induced by short amino acid stretches present in their protein sequences [14]. More examples for this phenomenon showed up [15,16,17] and it became clear that translocation through eukaryotic cell membranes can be mediated by short cationic or amphipathic peptides [18]. Due to that finding the rational design of CPPs was rendered possible [19], since some structure activity relationships of successful CPPs were figured out [20]. These achievements led to the development of chimeric CPPs consisting of sequence motifs derived from protein sources combined with rationally designed sequence motifs as well as the creation of completely artificial peptides [21], which proved to be membrane active [22]. CPPs, moreover, showed to be able to efficiently carry molecular cargo [23,24] through cellular membranes, a finding that established a practical relevance in biomedical applications for this class of molecules [25]. Cargo molecules selected from different entities, such as small probes [26], proteins [27,28], oligonucleotides [29,30], or nanoparticles [31] proved to be transportable. CPPs were found to be able to carry cargo that was larger in molecular size than the CPP itself into living cells. An issue in these transport events can be unwanted entrapment of CPP/cargo complexes in endosomes leading to lysosomal degradation rather than to delivery into the cytoplasm [32]. Imaging techniques were successfully applied to the visualization of transport processes of cargo molecules across multiple membranes in plant cells [33] and successful delivery of macromolecular cargo [34].

## 3. Uptake Mechanisms of Cell Penetrating Peptides

Despite intense efforts being executed to evaluate the molecular mechanisms of the internalization process [35], the mode of entry of CPPs into eukaryotic cells is still under discussion [36,37,38]. Structurally different CPPs are internalized via different mechanisms [39], the peptide concentration outside the cell and the composition of the lipid membrane are important parameters in these processes [40]. Translocation of CPPs into cells occurs by endocytosis in many cases, which has been investigated by testing the influence of endocytosis inhibitors, such as wortmannin on uptake efficiency [20]. Endocytic pathways include clathrin/caveolae dependent/independent models as well as macropinocytosis. These routes play major roles in many internalization processes [41], the rate of fluid phase endocytosis, or macropinocytosis might be directly influenced by the uptake of CPPs [42]. Cationic arginine rich CPPs were found to be able to transport conjugated molecular cargo by efficiently binding to proteoglycans on the cell surface [43]. This substantiates the finding that the mode of entry depends on the structural characteristics of the peptides; arginine rich peptides differ from amphipathic candidates [44]. Other mechanisms, such as the uptake via extracellular heparin sulfates or direct translocation seem to be involved [35]. Endocytosis independent mechanisms include toroidal pore, barrel stave pore, inverted-micelle, and carpet models. Beyond that, receptor mediated uptake is discussed, e.g., for oligonucleotides conjugated to CPPs, in this case, scavenger receptor A conveys uptake via endocytosis [45].

## 4. Antimicrobial Peptides

Natural AMPs are a relevant part of the innate immune system of multicellular organisms and constitute an effective protection against pathogenic microbes. The vast majority of AMPs are cationic, amphiphilic, or at least hydrophobic; the essential target structure of their biological activity is the membrane of the pathogen [46]. The latter property may explain why it is “surprisingly improbable” [46] that resistance to AMPs occurs. Due to the emerging crisis of antibiotic resistance [47], AMPs are of particular importance as a low-resistance alternative in therapy.

The class of cecropins presented by Bomann in 1981 [48] is considered the first description of AMPs isolated from nature. After a number of classes of natural AMPs were identified and isolated in the 1980s, among them defensins [49] and magainins [50], AMPs were categorized into different classes according to their molecular structure. The conventional classification is based on the number of cysteines or disulfide bridges [51,52]: Without bridges, the peptides are linear and often form α-helices, an example is cecropin. With one disulfide bridge, a cyclic peptide exemplified, e.g., by bactenecin results [53]. If a peptide has several disulfide bridges, a β-sheet structure is obtained, a prominent example being the class of defensins. Linear peptides with an accumulation of certain amino acids, for example proline or arginine, form another class [51]. Proline-rich AMPs (PrAMPs) are membrane permeable peptides able to act on intracellular targets, such as chaperone protein DnaK or the 70s ribosome [54]. Therefore, the essential target of this class of AMPs is not the membrane itself, but membrane permeation is an inevitable feature of these peptides to reach their actual target. The first member of the class of PrAMPs was the apidaecin from the honeybee [55], many of these peptides contain a proline/arginine/proline (PRP) motif in their amino acid sequence and consist of two sequence motifs, a conserved one for antibacterial activity and a variable one for antibacterial specificity.

Intracellular target structures are addressed by plant defensins; additionally, they act, e.g., by inhibiting α-amylase or other digestive enzymes [56]. Structurally, plant defensins consist of a triple stranded β-sheet structure connected to a α-helical sequence motif via conserved disulfide bridges. According to the connecting pattern of disulfide bridges, the class of defensins is divided into cis- and trans-defensins. The antifungal activity of AMPs was found to be mediated by the interaction with intracellular target structures as well. These interactions induce the generation of reactive oxygen species intracellularly leading to apoptosis of fungal cells [57].

Today, there are more than 3100 known AMPs of natural origin [58,59]. In comparison, there are more than 15,000 different synthetic peptides that show antimicrobial activity and are therefore also referred to as AMPs. To distinguish synthetic AMPs from natural AMPs the latter are also called host defense peptides (HDPs). The activity spectrum of AMPs is targeted against bacteria, fungi, viruses, parasitic protozoa, or against cancer cells [59,60]. The selectivity for distinguishing self- and non-self cells is mediated by the lipid composition of cell membranes [61,62]. This selectivity has been systematically overestimated for a long time due to different experimental conditions in determination of hemolysis and minimal inhibitory concentration [63]. Ultimately, the selectivity of a peptide is determined by its structure, which enables specific optimization towards distinct target structures on respective cells [64,65,66].

Regarding the biomedical applicability of optimized AMPs, their therapeutic potential in the body in particular is limited by their proteolytic degradability. Some pathogens even produce proteases on purpose, optimizing their survival strategy by degrading AMPs [67]. Targeted replacement of L-amino acids with D-amino acids in the peptide sequence of AMPs may lead to increased protease stability [68], since the presence of D-amino acids at cleavage sites prevents degradation by proteases, as already reported by Fridkin in 1990 [69]. This phenomenon is observed in other classes of membrane active peptides as well [70]. In addition to the need to increase in vivo stability, limitations in the difference between bactericidal activity and cytotoxicity are the reason that large efforts are required to bring peptides into clinical use via topical formulations [71]. Beside the antimicrobial activity of the AMPs, a second beneficial effect has been described: binding of AMPs to the LPS of gram-negative bacteria can lead to a neutralization of its endotoxic activity [72]. Classic topical formulations are creams [73] and droplets [74]. For systemic formulation of AMPs, an effort is ongoing to encapsulate them into nanocarriers, such as liposomes [75]. Nebulization of encapsulated peptides [76] was also successfully established.

## 5. Membrane Interaction of Antimicrobial Peptides

The membrane interaction of AMPs is determined by the structure-mediated properties of the peptide [77] and is apparently individual and diverse. Initially, the association and binding to the membrane occurs. In most cases, binding is mediated by nonspecific electrostatic interaction, and more rarely by hydrophobic or receptor-mediated interaction [78]. This is why local membrane properties, such as charge, curvature, membrane tension, and membrane asymmetry are usually more significant than individual properties of the lipid molecules. Depending on their concentration, the aggregation of the peptides [79] or insertion into the hydrophobic part of the membrane [80] may occur. The consequence is a modification of membrane properties; most important is a change in membrane permeability by pore formation or by interference with membrane integrity, which results in the compromise or killing of pathogens [81].

**Figure 1:**
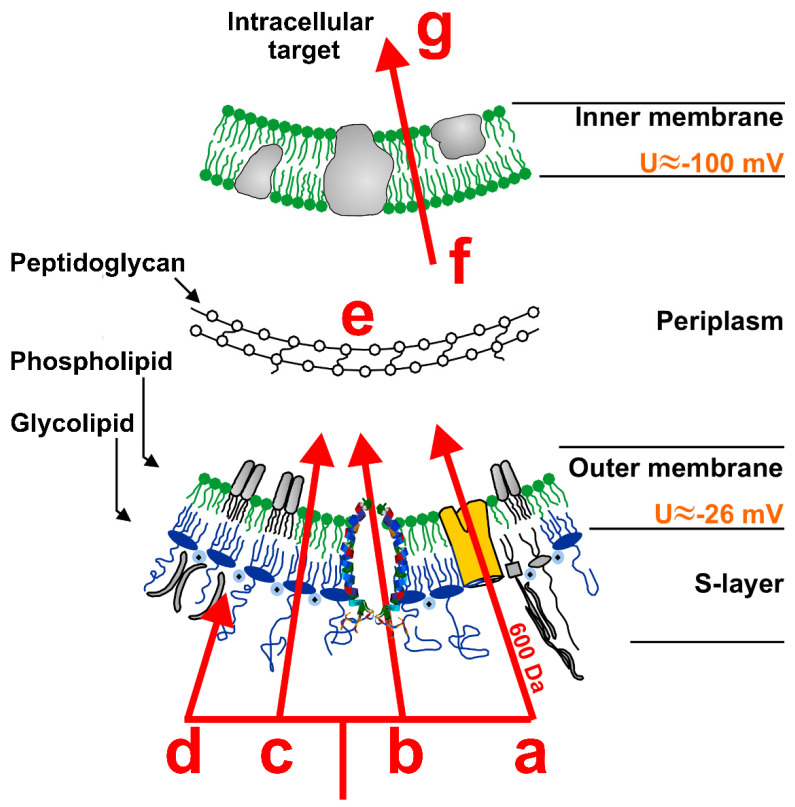
Possible interaction mechanisms of membrane active peptides illustrated on the example of the cell envelope of Gram-negative bacteria. The first barrier is the Outer Membrane. Peptides might (**a**) permeate through bacterial proteins up to a molecular weight limit of 600 Da; (**b**) induce lesions or pores allowing peptides to permeate through these self-formed apertures; (**c**) permeate directly through the lipid bilayer; or (**d**) bind to the membrane inducing changes in membrane properties. The second barrier is (**e**) the peptidoglycan layer and the last barrier is (**f**) the cytoplasmic membrane. If the permeabilization of the Outer- and Inner Membrane is sufficient for degrading microorganisms or if an Intracellular Target (**g**) needs to be attacked is still under debate. Since cell penetration peptides (CPPs) and antimicrobial peptides (AMPs) both do not have a well-defined target structure, optimization processes of these compounds are more complex on the one hand, but on the other hand the risk of resistance induction is low.

In 1974, for alamethicin, Baumann and Mueller described the formation of pores which were entirely lined by peptides oriented axially to the membrane surface [82]. This supermolecular alignment was later referred to as barrel stave model, and it turned out to be hardly transferable to any other peptide besides alamethicin [77,83]. In contrast to that, the toroidal-pore model has been attributed to a series of peptides. This model is based on a continuous curvature of the membrane surface, leading to an even lining of the pore by peptides and by lipid headgroups [84]. In 1992, the carpet model was proposed for dermaseptin S [85]: the peptides are aligned in parallel to the membrane surface, interact with each other, and lead to membrane destabilization that can culminate in a detergent-like breakdown of membrane integrity [86]. Based on these three prominent models, a variety of other membrane interaction models have been described (Figure 1). One of these is the sinking raft model, which is characterized by an axially asymmetric coverage of a lipid bilayer with peptides [87]. It builds on the carpet model and has been described for both α-helical and β-sheet peptides [88]. The more precise the lateral and temporal resolution of the membrane interaction of AMPs can be determined, the more doubt can be raised about the assignment of peptides to archetypal modes of action. However, these models provide useful ideas on real modes of action and can be used for their classification.

## 6. Optimization of Membrane Active Peptides

### 6.1. Computational Methods for Optimization of Membrane Active Peptides

#### 6.1.1. Machine Learning (ML) and Empirical Methods

Computational design and optimization of membrane active peptides is executed by various strategies [89]. These include pure in silico approaches, such as molecular dynamics simulations [90,91] or ML procedures based on, quantitative structure–activity relationship (QSAR) computational models [92] or support vector machines [93]. These methods aim to predict the potential of candidate peptide sequences to act as membrane active peptides before synthesis in order to save resources by preventing the synthesis of less-promising candidates [94]. Prediction of the properties of candidate peptide sequences in these approaches is based on information from databases or virtual libraries [95], such as APD3 [59] or CAMP [96] for AMPs (Table 1).

These data can be used to predict the activity of peptide candidates in de novo design strategies as well [97]. Structural parameters, such as sequence length, frequency of certain amino acid residues in the sequence, hydrophilicity, and overall charge of the peptides are linked to antimicrobial activity and used for the prediction of promising candidate sequences [98,99]. The linguistic model, for the first time, considered the linear arrangement of amino acid residues in a peptide sequence as some kind of language that follows physicochemical “grammar” rules. In that model, the properties of membrane active peptides are described by patterns of succeeding amino acid residues [100]. Insertion of membrane active patterns into amino acid sequences was successfully applied to the generation of peptides with improved membrane activity [101]. This concept was advanced by using natural templates of membrane active peptides for the generation of new artificial sequences with enhanced characteristics [102]. Some of the above-mentioned algorithms are freely available web-based applications that can be used in attempts to optimize AMPs based on QSAR criteria [103,104]. A combination of different algorithms allows the accurate identification of membrane active peptides in chemical space [105] requiring basic input of sequence data in FASTA format only. Beyond that, membrane active peptides selectively lysing the membranes of cancer cells are designed using ML techniques in the same way [106].

#### 6.1.2. Stochastic Methods

Another promising strategy for optimization of membrane active peptides is the use of evolutionary or genetic algorithms (GA) [107] that mimic “Darwinian” evolution in silico. Sequence information from lead peptides is recombined and point mutated according to fitness functions in order to generate filial peptides with improved characteristics. These algorithms are able to operate by using experimental data in their fitness functions. In principle, GAs are also able to use “Lamarckian” input in the form of information from databases or based on molecular descriptors [108] of a respective amino acid building block that characterizes its influence on membrane activity of a peptide. A thorough evaluation of the relevance of this information for particular optimization problems is mandatory [109], a finding which is related to the fact that not all descriptors available are suitable for delineation of the peptide membrane interaction under investigation. In particular, this applies to the descriptor-based optimization of CPPs, because in this class of membrane active peptides the relationship of sequence, structure, and function sometimes is difficult to define and to specify in descriptors.

GAs driven by experimental data can be susceptible to the effects of labels and tags, for example, penetratin is altered regarding its three-dimensional structure if labeled with different fluorophores [110]. These structural alterations in most cases are not sufficiently characterized regarding their influence on membrane activity. Therefore, within a few rounds of evolutionary optimization, a label or tag might become part of the molecule’s functionality. Here, a hardheaded view that a tag simply must remain a tag, thus different from the molecule of interest, might not withstand reality testing. This issue might be addressed by using different tags for labeling in parallel or testing the peptides functionality in label free assays. Another trap in GA-based optimization are mutually exclusive demands. If, for example, membrane activity occurs at high toxicity levels only in a population of lead peptides under investigation, optimizing for high membrane activity along with low toxicity might lead to a dead end in optimization, but illustrates that toxicity and transport activity are linked somehow. Successful convergence of a diverse set of lead peptide sequences to a consensus motif that represents a local fitness optimum in a reasonable number of generations requires a careful balance of mutation and recombination rates [111]. The use of GAs allow the simultaneous optimization of different molecular properties of membrane active peptides in parallel by considering multiple parameters for molecular evolution [112].

#### 6.1.3. Data Driven Algorithms vs. Stochastic Methods

Despite a diverse set of computational tools and algorithms applied to the design of AMPs for clinical application, so far, no computationally designed peptide candidates have reached advanced clinical trials, despite there being various AMPs derived from other sources under investigation (Table 2). This might be related to the fact that computational strategies used to optimize membrane active peptides rely on comprehensive training datasets. These data have to be appropriate for a particular optimization problem in order to yield meaningful optimization results. This finding applies to the design off CPPs as well, but in that case, the lack of knowledge regarding the mode of entry of these peptides into cells limits the reliability of training data used for computational design.

**Figure 2:**
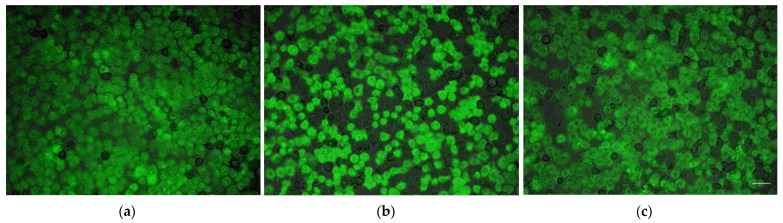
Transfection of monolayers of HeLa cells by different CPPs: (**a**) Fam-ALWKTLLKKVLKAPKKKRKV; (**b**) Fam-WLRRIKAWLRRIKALNRQLGVAA; (**c**) Fam-RLWRALPRVLRRLLR. Assay system to test the transfection efficiency of CPPs labeled n-terminally with 5-(6)-Carboxyfluorescein (Fam). Cells cultivated in 96-well µ-plates (ibidi, Munich, Germany) were incubated for 90 min at 37 °C with 10 μM solutions of peptide in culture medium. Overlay of fluorescence- and bright field images, images were acquired using a 10×/0.45 objective (Zeiss, Jena, Germany), a FITC filterset (ET480_40×/ET535_50 m/T510LPXRXT, Chroma, Rockingham, NC) and LED or oligochrome light source (FEI). Bar: 50 µm.

The design of membrane active peptides, therefore, might be more straightforward using methods that rely on trial and error based systems, such as GAs, rather than on data driven methods as represented by the above-mentioned ML algorithms. At that point, the validity of biological assay systems used to rank peptide candidates by fitness functions in this kind of algorithms is crucial. In an attempt to optimize CPPs, a genetic algorithm lacking any input from databases was applied [113]; only laboratory data from experiments performed under strictly comparable conditions were used for the determination of the fitness values of the different candidate CPPs. The fitness values of all lead peptides used were obtained in the same way in order to ensure a reliable fitness ranking [114]. For that, sequence information of roughly 500 peptides was extracted from a database [115], but all peptides were again synthesized in parallel and tested in a cell-based assay (Figure 2). All peptides were used in the same concentrations; the cells were incubated for the same time with the candidate peptides and, importantly, cells from one and the same batch were used in the experiments to ensure comparability [116]. On the contrary, in most databases, literature data are collected that were generated under non-standardized conditions, an issue which prevents these datasets from being useful for ranking of peptide candidates for optimization by computational methods, such as GAs.

### 6.2. Combinatorial Synthesis Methods to Optimize Membrane Active Peptides

Another way to identify membrane active peptides is to screen peptide libraries for active candidates [117,118,119,120]. In these efforts, a large number of peptides need to be synthesized in small amounts in order to investigate their membrane activity [121,122]. This can be achieved by synthesizing nanomolar amounts of peptides on solid supports [123] following a rational library design approach or in a combinatorial approach, as implemented by split and mix synthesis of picomolar amounts of peptides [124,125]. Split-split methods can be used to systematically shape the sequence space in one bead one compound libraries [126], this way short AMPs were optimized regarding their hemolytic activity. Rational combinatorial libraries are a compromise between the above-mentioned library designs and were applied successfully to the identification of AMPs with pore forming activity [88]. In these screening methods, a tradeoff between the detailed characterization of peptide candidates in small libraries and a perfunctory inspection of putative membrane active peptides in large combinatorial libraries has to be accepted.

### 6.3. Assay Systems for Characterization of Membane Active Peptides

The computational design of membrane active peptides demands a profound knowledge of molecular properties that are required for biomedical applicability of the peptide candidates. This conclusion leads to an awareness regarding the inalienability of reliable assay systems that enable the characterization of membrane active peptides’ properties. Any bias from these assays will hinder the multi-objective design of membrane active peptides. This is due to the fact that the respective assay results act as optimization criteria in design methods driven by experimental data. Therefore, any assay systems used have to reflect the requirements of biomedical application at its best.

#### 6.3.1. Membrane Activity Assays

Beyond several common pharmacological parameters, such as low toxicity, in vivo stability, and adequate water solubility, in particular, the membrane activity of the candidate peptides has to be investigated. Since there are no theoretical models that are able to predict the membrane activity of all peptide sequences conceivable, biological assay systems are required that enable the proper evaluation of the peptides’ activity. For optimization purposes, all candidate peptides have to be evaluated in the same assay system, since standardization of data from different assays is difficult.

Membrane activity of CPPs is usually analyzed in vitro (Figure 2). Cells are grown in microtiter plates and transfection of cell monolayers is monitored via optical measures, such as intracellular fluorescence [114] in parallel for multiple peptide candidates. It is important to keep the parameters for uptake of different peptides into cells sufficiently similar. The membrane activity quantified in these assays is influenced not only by defined experimental conditions, such as temperature and peptide concentration, but also by cell-specific factors, such as confluence level and growth characteristics of the cells used. Moreover, as visualized in Figure 2, not all cells in a monolayer might be transfected by a particular cell-penetrating peptide, this is an important observation and has to be considered when analyzing data from cell-based assays. In vitro assay systems employed in the literature to analyze the performance of CPPs and issues of these methods are reviewed elsewhere [127].

Membrane activity of AMPs is characterized by assays that test the antimicrobial susceptibility of the peptides. Classically, the concentration of an AMP is determined, which inhibits bacterial growth. This minimum inhibitory concentration (MIC) is the most important metric in the characterization of an AMP. Another important parameter that describes the suitability of an AMP in biomedical application is its hemolytic activity [128]. Methods were established that aim to reflect clinically relevant parameters, such as the influence of biological matrices on the efficacy of AMPs or deviations in antimicrobial potential regarding different target microbes [129]. The interaction of AMPs with bacteria can be measured in a high-throughput assay utilizing peptide arrays on membrane supports [130] to be able to compare large numbers of peptide candidates.

#### 6.3.2. Assays Quantifying other Optimization Criteria

Biomedical application of membrane active peptides requires a multi-objective optimization of peptide candidates, because absorption, distribution, metabolism, and excretion (ADME) criteria, among others, are important features in preclinical and clinical settings beyond membrane activity. These parameters are monitored by assay systems because computational determination of these features [131] is an important tool in preclinical environments, but is to date not fully approved in clinical trials [132]. ADME criteria, such as metabolic stability of therapeutic peptides, have to be optimized [133] since the in vivo stability of membrane active peptides is a critical parameter regarding their biomedical applicability. Proteolytic cleavability of peptides can be assessed by mass spectrometry [134] in serum or plasma samples or by ELISA-based methods [135] in mucosal secretions. These kinds of assays are particularly susceptible to sample preparation issues since matrix effects of bystander proteins may dampen the proteolytic degradation of the peptide of interest drastically. This means that the in vivo situation is not always the worst case regarding proteolysis but, for example, in a plasma sample a peptide might be more prone to digestion than in whole blood [134]. Determination of half-live of therapeutic peptides in serum or plasma might therefore be misleading with regard to the in vivo stability of the respective peptide candidates. The proteolytic stability of peptides can be altered by incorporating non-natural amino acid building blocks into the sequence or by grafting active peptides onto molecular scaffolds [136].

## 7. Discussion

The design of membrane active peptides requires a multi-objective approach that considers membrane activity on eukaryotic lipid membranes for CPP and activity on bacterial envelopes for AMP candidates. In biomedical applications, the membrane activity is a necessary but not a sufficient characteristic of a candidate peptide. ADME criteria, such as toxicity, immunogenicity, and metabolic stability in vivo are equally important features of a membrane active peptide that have to be considered in optimization attempts. In particular, bacterial resistance is another important point to consider. On the one hand, the peptides must overcome naturally occurring resistances; on the other hand, new resistances must be prevented. Depending on the formulation in which the peptides are to be used, as a cream, tablet, injection, or coating of implants, optimization parameters must be adapted.

The computational design of membrane active peptides using ML methods driven by information from databases is complicated by the fact that the interaction of membrane active peptides with lipid membranes is not fully understood so far and seems to be dependent on a wide range of parameters, such as temperature, peptide concentration, and composition of the membrane. Therefore, stochastic approaches, for instance genetic algorithms driven by information from customized biological assay systems, seem to be even more promising tools to design novel membrane active peptides. These compounds are urgently required in different biomedical applications, such as in drug delivery or as novel antimicrobial agents.

## Figures and Tables

**Table 1:** Databases (all accessed on 6 January 2022) containing information on antimicrobial peptides (AMPs).

Database	Number of Entries	Content	Hyperlink
LAMP2	23,253	AMPs, structure, collection, composition, source, function	http://biotechlab.fudan.edu.cn/database/lamp/index.php
DRAMP	22,259	AMPs, structure, activity physicochemical-, patent-, clinical data	http://dramp.cpu-bioinfor.org/
DBAASP_v3.0_	17,865	AMPs, structure, activity	https://dbaasp.org/
CAMP_R3_	8164	AMPs, Structure, patents, signatures	http://www.camp.bicnirrh.res.in/
Cybase	4012	Cyclic proteins, antiviral, insecticidal, antibacterial	http://www.cybase.org.au/
APD3	3324	AMPs, structure, activity	https://aps.unmc.edu/
DAPD	2571	Structure, activity, host taxonomy	http://split4.pmfst.hr/dadp/?
YADAMP	2525	Structure, activity	http://yadamp.unisa.it/default.aspx
DAMPD	1232	taxonomy, species, AMP family, citations	http://apps.sanbi.ac.za/dampd/
AntiTbPdb	1010	Anti-mycobacterial peptides, structure, activity	https://webs.iiitd.edu.in/raghava/antitbpdb/
InverPep	702	Invertebrate AMPs, structure, activity, target	https://ciencias.medellin.unal.edu.co/gruposdeinvestigacion/prospeccionydisenobiomoleculas/InverPep/public/home_en
ANTISTAPYBASE	596	AMPs, structure, activity against MRSA	https://www.antistaphybase.com/
Defensins	363	Structure, activity	http://defensins.bii.a-star.edu.sg/
Peptaibols	317	Fungal AMPs, non-standard amino acids	http://peptaibol.cryst.bbk.ac.uk/home.shtml
PhytAMP	273	Plant AMPs	http://phytamp.hammamilab.org/main.php
BACTIBASE	230	Bacteriocins, structure, function	http://bactibase.hammamilab.org/about.php
BaAMPs	221	Biofilm active AMPs	http://www.baamps.it/
THIOBASE	39	Thiopeptides, structure, activity	https://bioinfo-mml.sjtu.edu.cn/THIOBASE/index.php
EnzyBase	N/A	Encybiotics, lysins, lysocymes, bacteriocins	http://biotechlab.fudan.edu.cn/database/EnzyBase/home.php
MBPDB	N/A	Milk bioactive peptides, function, species	http://mbpdb.nws.oregonstate.edu/

**Table 2:** Membrane active peptides under investigation in clinical trials.

Name ^1^	Conditions	Description
TAPS-18	Periodontitis	Cathelicidin based synthetic peptide
LEAP2	Type 2 Diabetes	39-mer synthetic liver expressed amp
LEAP2	Obesity	39-mer synthetic liver expressed AMP
MSI78	Diabetic foot infection	broad-spectrum synthetic analogue of magainin
LTX109	Skin Infection, MRSA Inf.	Synthetic peptidomimetic
LL37	Melanoma	Cathelicidin based
hlf1-11	Bacterial Infections, Mycoses	Lactoferrin derived
PLG0206	Joint Infection	Engineered AMP
Pxl01	Surgical adhesions	Lactoferrin derived
IB367	Pneumonia, mucositis	synthetic analogue of Protegrin I
Pac113	Oral Candidiasis	Histatin derived
MX-594AN	catheter-related acne	Indolicidin based
rBPI21	meningococcaemia	human bactericidal permeability protein derivative
ETD151	fungal infections	44 mer variant from lepidopteran Heliothis virescens
HB-50	anti-infective	synthetic natural peptide mimetic of cecropin
HB-1345	broad-spectrum antibiotic	Synthetic Lipohexapeptide
CZEN-002	vulvovaginal candidiasis	synthetic 8-mer from α-melanocyte-stimulating hormone
PTX005	antimicrobial	Synthetic 12 mer
Glutoxim	Tuberculosis, NSCL cancer	thiopoietin
IMX942	Nosocomial infections	Synthetic cationic host defense peptide
NP213	Fungal infections	cyclic cationic peptide from NovaBiotics arginine peptide platform
OP-145	Chronic bacterial middle ear infection	Synthetic 24-mer peptide derived from LL-37
CD-NP	Organ failure	Synthetic chimeric 37-mer
C16G2	Treatment of dental subjects	synthetic AMP
Sifuvirtide	HIV fusion inhibitor; AIDS	designed based on the 3D structure of the HIV-1 gp41
POL7080	nosocomial pneumonia	synthetic by amino acid substitution of protegrin I
Omiganan	atopic dermatitis, rosacea	Synthetic 12-mer cationic peptide derived from indolicidin

^1^ Denomination of compound in clinical trial

## Data Availability

Not applicable.
